# Biological interpretation of deep neural network for phenotype prediction based on gene expression

**DOI:** 10.1186/s12859-020-03836-4

**Published:** 2020-11-04

**Authors:** Blaise Hanczar, Farida Zehraoui, Tina Issa, Mathieu Arles

**Affiliations:** IBISC, Univ Evry, Université Paris-Saclay, 23 boulevard de France, 91034 Evry, France

**Keywords:** Deep neural network, Biological interpretation, Phenotype prediction

## Abstract

**Background:**

The use of predictive gene signatures to assist clinical decision is becoming more and more important. Deep learning has a huge potential in the prediction of phenotype from gene expression profiles. However, neural networks are viewed as black boxes, where accurate predictions are provided without any explanation. The requirements for these models to become interpretable are increasing, especially in the medical field.

**Results:**

We focus on explaining the predictions of a deep neural network model built from gene expression data. The most important neurons and genes influencing the predictions are identified and linked to biological knowledge. Our experiments on cancer prediction show that: (1) deep learning approach outperforms classical machine learning methods on large training sets; (2) our approach produces interpretations more coherent with biology than the state-of-the-art based approaches; (3) we can provide a comprehensive explanation of the predictions for biologists and physicians.

**Conclusion:**

We propose an original approach for biological interpretation of deep learning models for phenotype prediction from gene expression data. Since the model can find relationships between the phenotype and gene expression, we may assume that there is a link between the identified genes and the phenotype. The interpretation can, therefore, lead to new biological hypotheses to be investigated by biologists.

## Background

Precision medicine consists of using genetic characteristics of patients in order to guide and improve clinical decision making such as diagnosis, prognosis, choosing the most appropriate treatment, etc. It has the potential to change medical practices profoundly. “Omics” technologies, such as genomics, transcriptomics (sequencing, microarrays) and proteomics (protein chips, tissue arrays), have significantly altered the scale of data and provided massive amounts of genomic data collected from patients. The variation in gene expression allows the study of complex pathologies. The use of classifiers, constructed from gene expression profiles in clinical research to assist decision making, is becoming more and more important. Machine learning methods including support vector machine, random forest and boosting are among the main tools used in making biological discoveries from the huge amount of available gene expression data [1].

Among the various machine learning approaches, deep learning has become one of the most powerful methods [2]. Its primary domain of application is image recognition and speech recognition where it has beaten other machine-learning techniques records. Deep learning algorithms are promising in many other domains of science, especially in precision medicine and genomics data analysis since it is very good at discovering intricate structures in high-dimensional data. However deep learning methods are still very new in the bioinformatics community, and few works have been published on its application to gene expression based classification [3]. Unlike images or text data, gene expression data has no structure that can be exploited in the architecture of a neural network. The architecture used for prediction from gene expression data is therefore the multilayer perceptron [4, 5]. The autoencoder is another architecture commonly used to reduce the dimensionality of the gene expression data, like the denoising autoencoders [6, 7] or variational autoencoders [8] . The idea is to use the middle layer of the autoencoder as a compact representation of the gene expression profiles that captures the useful biological information for the prediction task. Fakoor et al. [6] used both a stacked autoencoder and principal component analysis (PCA) in order to reduce the dimension of the data before constructing a neural network for cancer prediction. Dincer et al. [9] first use PCA then, a variational autoencoder for the dimensionality reduction and a LASSO to predict drug response to leukemia. Hanczar et al. [10] use a multi-layer perceptron to predict cancers and pretrain each layer with a denoising autoencoder and a large unlabelled dataset.

One of the main concerns of deep learning in medical applications is its lack of interpretability. Neural networks can be viewed as black boxes, where the gene expression profile of a patient is put to its input layer and a prediction is obtained from its output layer without providing any explanation on the decision process. The need for making deep neuronal networks more interpretable is therefore increasing, especially in the medical field for mainly two reasons. Firstly, it is important to ensure that a neural network bases its predictions on reliable representations and is not focusing on an artifact of the data. Without the interpretability criterion met, physicians cannot trust the decision of the neural network and patients’ lives may be at stake. It is crucial to be able to identify which neurons, genes and related biological processes are involved in the prediction and decision making process. Secondly, a neural network with high prediction performances may have identified patterns in the gene expression that could lead to new biological hypotheses. To investigate these patterns it is crucial to understand what is the biological meaning of the hidden layers of the network.

The interpretation of machine learning algorithms is still an emerging field of research especially for deep learning models [11, 12]. Two types of interpretation may be identified [13, 14]: prediction interpretation and model interpretation. The prediction interpretation consists of explaining the prediction of a specific input, whereas model interpretation explains the logic behind the model when predicting the different outputs on the whole population. Both are important for medical applications. The interpretation of neural networks built from gene expression has not been thoroughly studied. The majority of the published works focuses on the identification of the genes that impacted the prediction but does not investigate the representation of the gene expression learned in the hidden layer. For example, Danaee and Ghaeinix [15] identified relevant genes for the diagnosis of breast cancer using stacked denoising autoencoders. The relevant genes are those with a strongly propagated influence on the reduced dimension of the network and are analyzed using the Kyoto Encyclopedia of Genes and Genomes (KEGG) and Gene Ontology (GO). The aim of all these studies is to identify potentially interesting genes related to the disease of interest. However, they do not explain what the network does, or what represents a neuron, or what representation of the patient is constructed in the hidden layers. Very few works tried to interpret the hidden neurons and almost all of them are based on the analysis of the values or the distribution of connection weights of the learned neural network [16]. Way et al. [8] analyzed the decoder’s connections of their variational autoencoder and associate each neuron to the set of genes with the highest absolute values of weight. Based on these gene sets, they applied an enrichment analysis to identify overrepresented pathways and GO biological process terms to each neuron. In [16], authors built denoising autoencoders and stacked denoising autoencoders to extract important genes from cancer gene expression dataset. The importance of genes is defined as the sum of their outgoing connections. A subset of the most important genes is selected and then analyzed by performing a functional annotation analysis. Sharifi et al. [7] studied the distribution of the output weights of each neuron in order to estimate their significance for the prediction of the metastatic tumor.

Recent works in the machine learning community show that the use of gradient methods produces better interpretations of a neural network than an analysis of their weights [17]. The principle of gradient methods is to backpropagate the activation of the output neuron through the network and to estimate for each layer the impact of the neurons and the connections on the output. Several gradient methods were proposed in the literature including layerwise relevance propagation (LRP) [18, 19], integrated gradients [20] and DeepLift [21]. In [22], the authors presented a unified framework for interpreting predictions by analyzing several gradient interpretation models from theoretical and practical perspectives. They showed that these methods are strongly related and equivalent under certain conditions. Lundberg and Lee [23] showed that among gradient models DeepLift and LRP are better aligned with human intuition as measured by studies since they satisfy some desirable properties. To our knowledge, only one paper used the integrated gradient method to identify the most important genes related to a low-dimensional representation space (LDR) learned using a variational autoencoder [9]. The main objective of our work is to open the black box of a deep neural network built from gene expression data by linking the neurons with biological knowledge. Our approach adapts gradient based approaches of neural network interpretation in order to identify the important neurons i.e. the most involved in the predictions. Then all these important neurons are associated with genes, biological functions and metabolic pathways. Our experiments on cancer prediction show that our approach produces more relevant interpretation than the state of the art based on weight analysis. Although the main purpose of this paper is not on the classification performance, we show that for large training sets, deep learning outperforms classical machine learning methods.

## Results

### Gene expression dataset

We applied our method to a cancer diagnosis problem extracted from microarray data. The used data comes from a study of cross-experiment compiling the gene expression profile from about 40,000 publicly available Affymetrix HG-U133Plus2 arrays [24]. Combining different expression datasets gives a global gene expression map that contains variability related to the type of tissues and the experimental protocols. This allows us to address new questions and to make original studies that may lead to new biological discoveries. After quality control and normalization, the dataset contains the expression of 54675 probes from 27887 tissues of which 9450 are healthy and 18437 are cancer. The dataset is accessible via the ArrayExpress database (accession number E-MTAB-3732). We divided this dataset into a training set (14750 cancer, 7560 non-cancer) and a test set (3687 cancer,1890 non-cancer). Because of the exceptionally large size of this dataset, it is not necessary to reduce its dimension by an autoencoder or a gene selection method.

### Neural network model

We constructed a deep multilayer perceptron with an input layer of 54675 neurons, three hidden layers of 500, 200, 50 neurons respectively and an output layer of two neurons corresponding to the non-cancer and cancer classes. To reduce the problem of overfitting, the dropout is used in the output of the first and second hidden layers ($p_{drop}$ is the probability to drop a neuron) and a L1 penalization term is added to the cross-entropy loss function ($\lambda$ is the hyperparameter controlling the weight of the penalization). The network is learned using the adam optimizer with a learning rate *lr*. The end of the training is controlled by an early stopping procedure with a maximum of 500 epochs. The hyperparameters are optimized on a validation set containing 10% of the training set. Table 1 gives the list of the hyperparameters, the range of tested values and the selected values. The experiment has been implemented using Tensorflow 1.12 and computations launched on a GPU Geforce TitanX.

**Table 1:** List of the hyperparameters optimized during the learning procedure

Hyperparameters	Selected values	Tested values
# hidden layers	3	1,2,3,4,5,6
# neurons	500/200/50	[20,1000]
$p_{drop}$	0.3	[0.1, 0.5]
$\lambda$	$10^{-3}$	$[10^{-2},10^{-5}]$
*lr*	$10^{-4}$	$[10^{-2},10^{-5}]$
batch size	16	[8, 256]

**Fig. 1:**
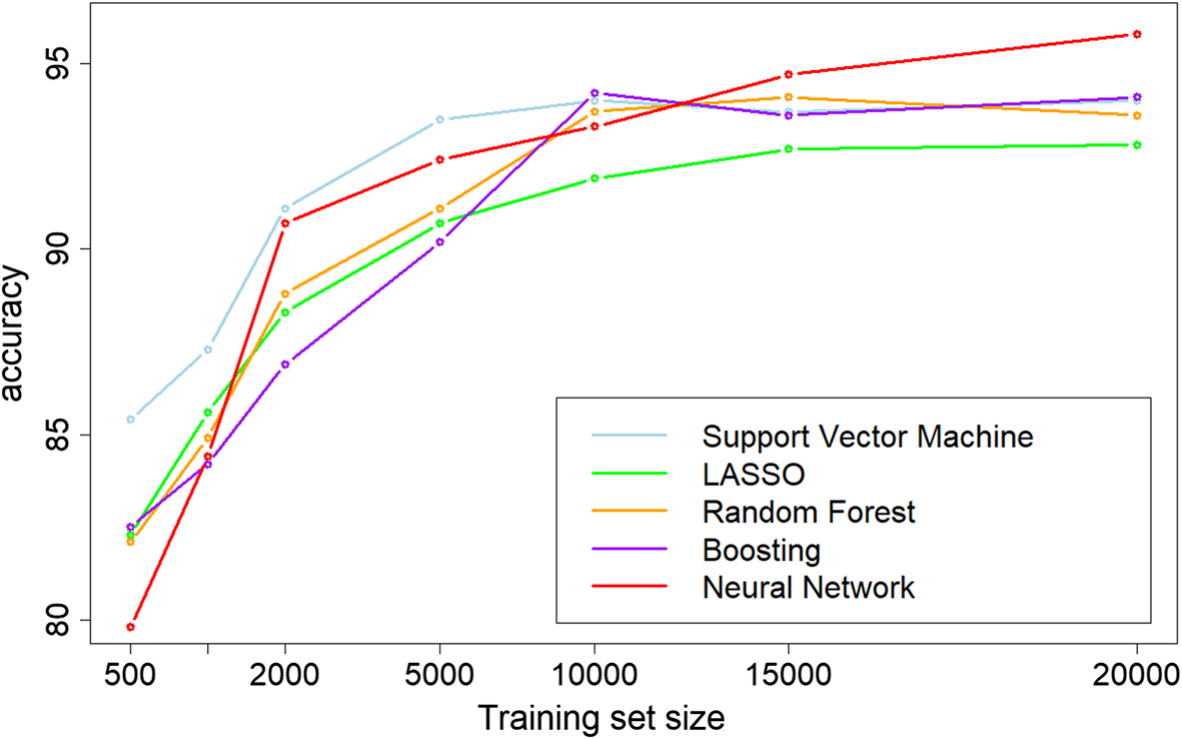
Accuracy of the different learning algorithms in function of the training set size

The performances of the neural network (NN) are compared with the state-of-the-art of supervised learning methods i.e. support vector machine (SVM), least absolute shrinkage and selection operator (LASSO), random forest (RF) and XGboost (XGB) by varying the number of available training examples *n*. We randomly selected subsets of the training set of different sizes by conserving the same proportion of the classes. For each training subset, classifiers have been constructed with different learning algorithms. Figure 1 illustrates the accuracy of the different classifiers depending on the training set size. We show that for small training set size ($n<1000$) the state-of-the-art algorithms provide higher accuracy than NN, for moderate training set size ($2000<n<10000$), the accuracy of NN is similar to the accuracy of the other methods, for large training set size ($n>15000$) the NN significantly outperforms the other methods. When the whole training dataset is used, the accuracy of the NN is estimated at 95.7% on the test set. Among the 238 prediction errors, 115 are non-cancer patients predicted as cancer and 123 are cancer patients predicted as non-cancer. This model will be analyzed in detail in the rest of the paper. We note that the performances of SVM, LASSO, RF and XGB reach their maximum for $n \in [10000,15000]$ examples, adding new training examples does not improve the accuracy. The accuracy of NN is still increasing for $p=20000$, we could reasonably assume that its accuracy will continue to improve if more training examples are available. These results are coherent with the results of NN from other domains (image analysis, NLP,...) i.e. NN are especially good when the training set is large. Currently, gene expression datasets are small, they contain generally hundreds of examples. This explains why today NN has not made a breakthrough in gene expression based classification yet. In the next years, with the increasing production of transcriptomic data, it is clear that NN will play a major role in these problems. That is why the interpretation of these models should be studied from now.

Shallow supervised machine learning methods like RF, SVM and XGB can be explained using approaches that extract the most important input features for the predictions. In this work, we focus on the interpretation of deep learning models by identifying the relevant input and high level features learned by the model.

### Relevance scores analysis

In order to interpret the learned model, the relevance vector of each example in the test set is computed. The relevance vector of an example contains the relevance score, computed by LRP, of all neurons of the network. Note that LRP is applied from the output neuron corresponding to the predicted class. A relevance vector represents therefore which part of the network is the most responsible for the prediction of a given example. An analysis of these individual relevance vectors shows that, for almost all of the examples, only a small set of neurons is important, i.e. have a high relevance score. However, the important neurons associated to two examples may be very different, even if the model assigns them the same class. For each class, we can identify different groups of relevance vectors, meaning that different parts of the network are specialized to predict from different groups of examples even if these examples belong to the same class.

**Fig. 2:**
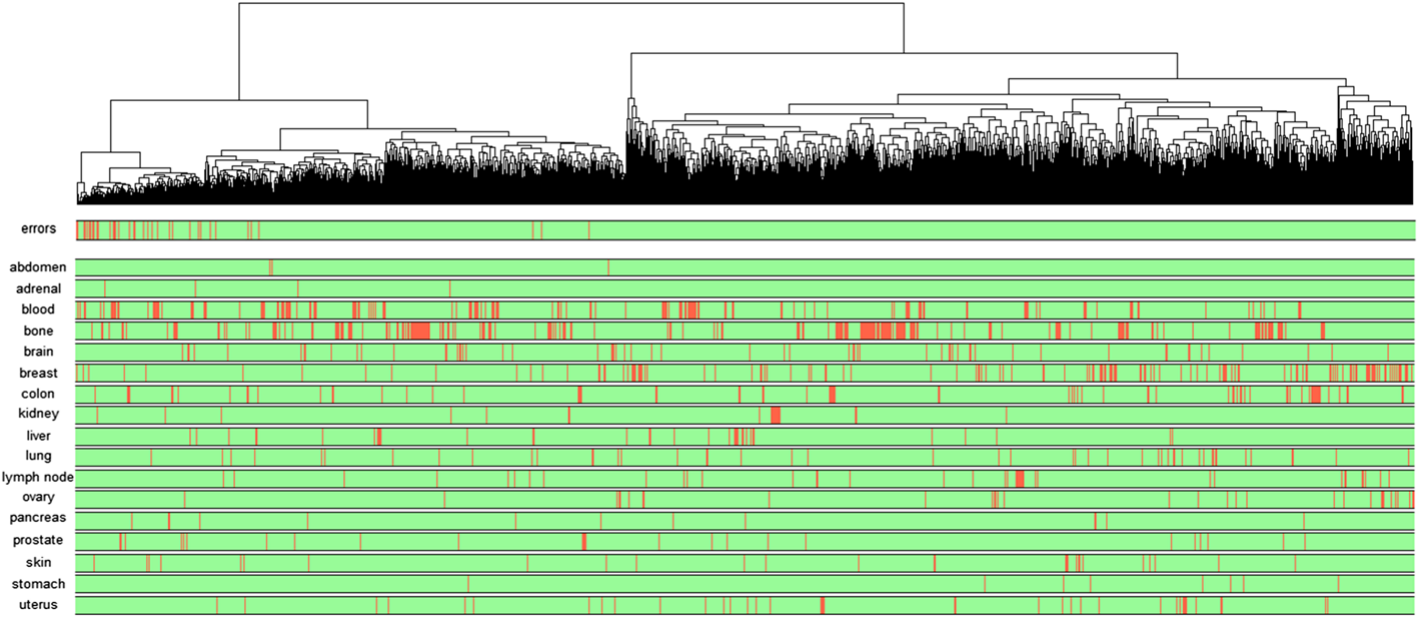
Hierarchical clustering of the test examples predicted as cancer based on their relevance vectors. In the first colored bar, red represents prediction errors and green corresponds to good predictions. The other colored bars show the type of tissue

Figure 2 shows the hierarchical clustering of the test examples predicted as cancer based on their relevance vectors. The first colored bar in the bottom gives the prediction result: green means that the corresponding test example is well classified and red means it is a prediction error. The nest colored bars gives the type of tissues. From this dendrogram, we see clusters of test examples with different relevance profiles. It’s worth noting that the clustering based on the relevance vectors does not overlap with the clustering based on the gene expression profiles. Examples with different expression profiles may use the same neurons for their predictions whereas examples with similar expression profile do not necessary have similar relevance vectors.

An interesting observation is that the prediction errors tend to be grouped in some clusters. This means that the error of predictions often comes from the same set of neurons. Therefore, the network contains some paths of propagation that lead to less confident predictions. If we look at the distribution of the examples on the dendrogram according to the type of tissue, we do not observe any particular pattern. The examples from the same tissue are not located in the same cluster. This means the way an example is propagated through the network does not depend on the tissue. Two explanations are possible. The first one is that the network has discovered a general signature of cancer for any type of tissue. We know that the different types of cancer have different biological causes, but a part of the information contained in the gene expression may be common to all cancers. The second explanation is that the network found different signatures for the different tissues but these signatures are merged into the same set of neurons. In this case, it could be interesting to modify the architecture of the network by adding auxiliary outputs predicting the tissue type from hidden layers, such that the different tissues use different neurons in the hidden layer. This idea will be investigated in future works.

### Comparison with WM interpretation

In the majority of NN interpretation works for gene expression, the evaluation of the impact of a neuron (input or hidden) is based on the weight average of their output connections (WM). However, in many situations, the WM score does not represent the real contribution of an input or neuron to the prediction.

**Fig. 3:**
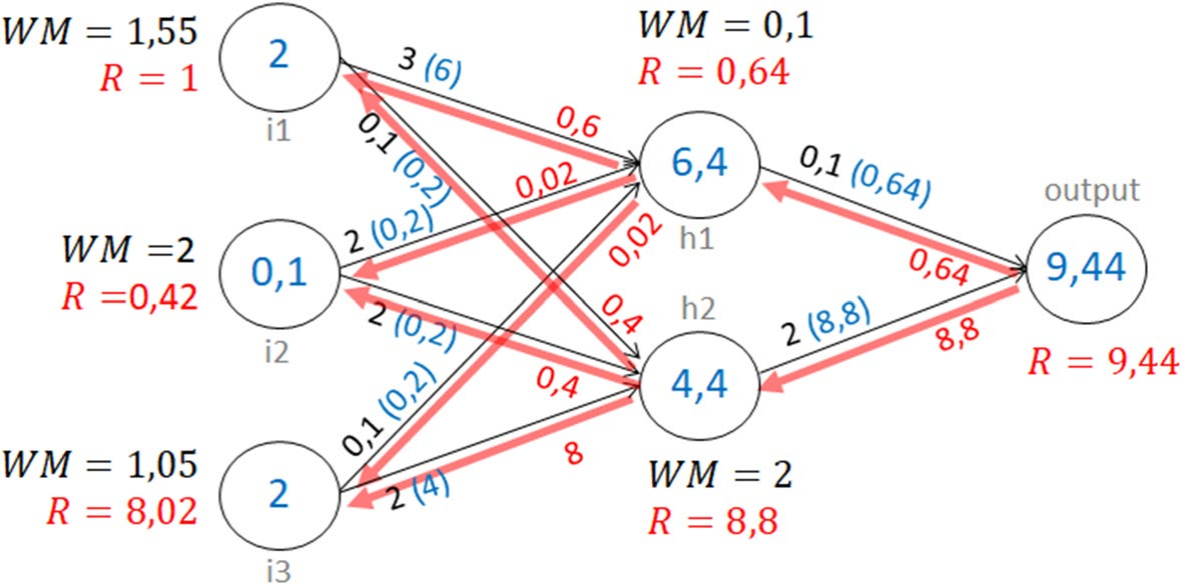
Comparison of LRP and WM on a toy example

Figure 3 represents a toy example illustrating the difference between the WM and LRP methods. We have a neural network with three layers containing three inputs (i1,i2,i3), two hidden neurons (h1,h2) and one output. The black arrows represent the connections with their weight. The blue numbers show the propagation of an example from the input ([2, 0.1, 2]) to the output ([9.44]). The importance of the input and hidden neurons is computed by WM method and represented in the figure in black. The LRP is also applied in order to back-propagate the activation of the output through the network and is represented by the red arrows. The red scores represent the relevances computed for each input and hidden neurons. This example points out the difference of interpretation obtained by the WM scores and LRP relevance. It shows that the WM values are not a good evaluation of the impact of the neurons on the output. According to the the WM values, i2 is the most important input because its connection weights are high. However, since the activation of i2 is low, its impact on the output is actually small. The WM value of i1 is 75% of the WM value of i2, leading to the conclusion that this input has a significant impact on the output. i1 is highly activated and this activation is propagated through h1 and h2. Since the weight of the connection i1-h2 is small, this path does not have a high impact on the output. The weight of the connection i1-h1 is leading to a high activation of h1. However, the weight of the connection h1-output is small, therefore h1 weakly activates the output. The impact of the input i1 on the output is thus small because its activation is not well-propagated to the output. The input i3 has the smallest WM value, we could conclude that its impact on the output is not significant. However i3 is highly activated and this activation is well propagated to the output thanks to the connections i3-h2 and h2-output that have high weight. In fact, the activation of the output comes mainly from i3. The relevance of i3 is very high and the relevance of i1 and i2 are small. The LRP relevances can be considered as a better representation of the real impact of the input on the output than the WM values since the latter is independent from the input data. By definition WM values are computed only from the weights of the connections. These values could represent the importance of the inputs if the variables were independent and uniformly distributed. However, this is not the case in the context of gene expression data. The interpretation must depend on the distribution of the data that are provided to the network.

**Fig. 4:**
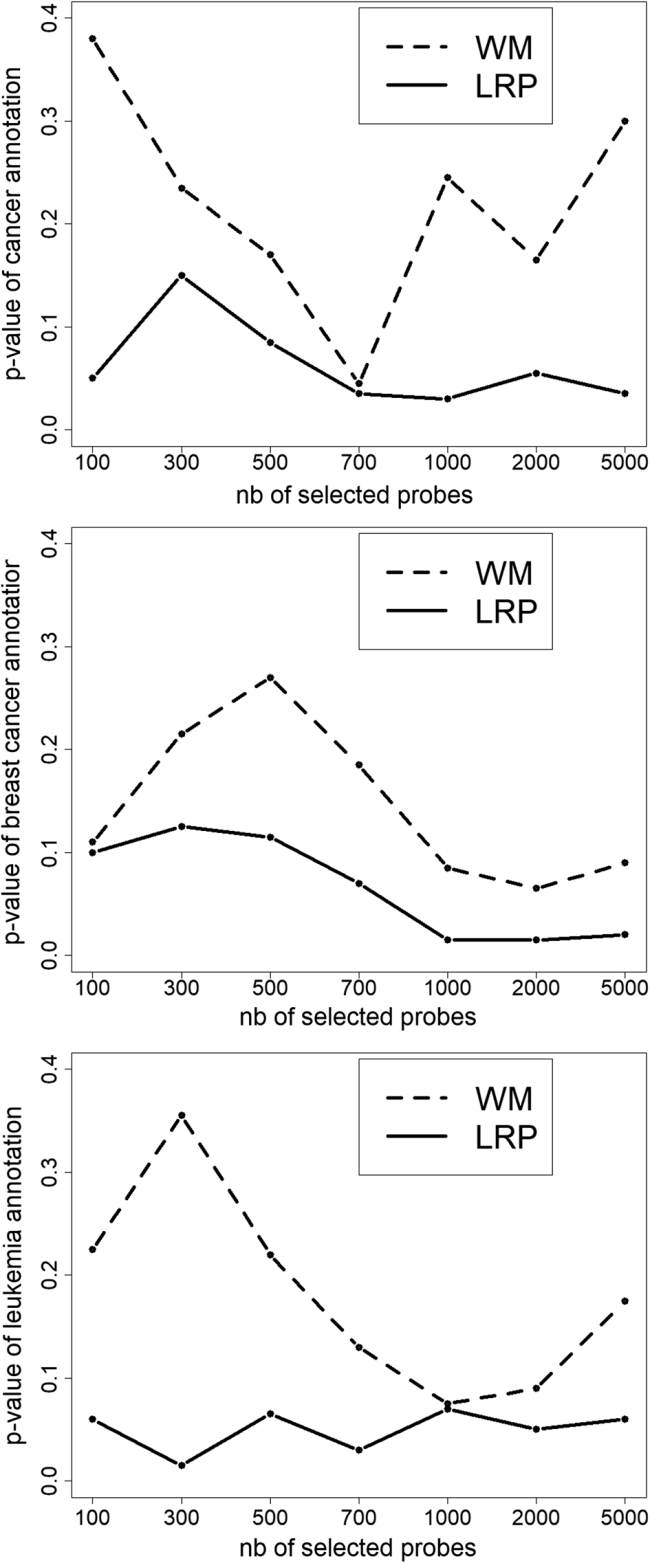
p-values of the terms: cancer, breast cancer and leukemia from subsets of genes selected by LRP and WM

We propose an experiment to compare the quality of biological interpretation produced by WM and the LRP on our gene expression dataset. In this experiment, we measure the consistency of the LRP and WM interpretations of our cancer prediction network with known biological knowledge. We use all the test examples predicted as cancer by our network to compute the average of the LRP relevance scores of the input neurons. The WM values of each input neuron are also computed. These results give, for each method, a ranking of the genes that have the most impact on the predictions of cancer in our network. Based on these rankings, the *k* best genes are selected ($k \in [100,5000]$). Then, an enrichment analysis is performed in the same way as described in section 2.3, in order to identify the over-represented diseases from DisGeNET associated with the selected genes. DisGeNET is one of the largest public collections of gene-disease associations. Figure 4 (top) shows the p-values of the cancer annotation obtained from these analyses depending on *k*. We see that the p-values obtained from the LRP selection are much smaller than the p-values given by WM selections. The obtained values are around 0.05, except for $k=300,500$. This shows that there is an over-representation of the genes linked to cancer in the set of genes with the highest impact on the prediction of cancer. In other words, these results show that our network mainly uses genes, that are known to be related to cancer, to make cancer predictions. The fact that the behavior of our network is coherent with the biological knowledge, tends to improve the confidence given to its predictions. The WM values do not lead to the same conclusions, the p-value of cancer is below 0.1 for only $k=700$. The other graphics of Fig. 4 show the results of the same experiments restricted to patients affected by breast cancer (middle) and leukemia (bottom). These results show that the LRP produces more relevant interpretation than WM, the LRP p-values are much lower than the WM p-values. WM is data independent, the produced interpretation is therefore very general. Since LRP computes a relevance score for each example, it leads to more relevant interpretations.

### Biological interpretation of the model

**Fig. 5:**
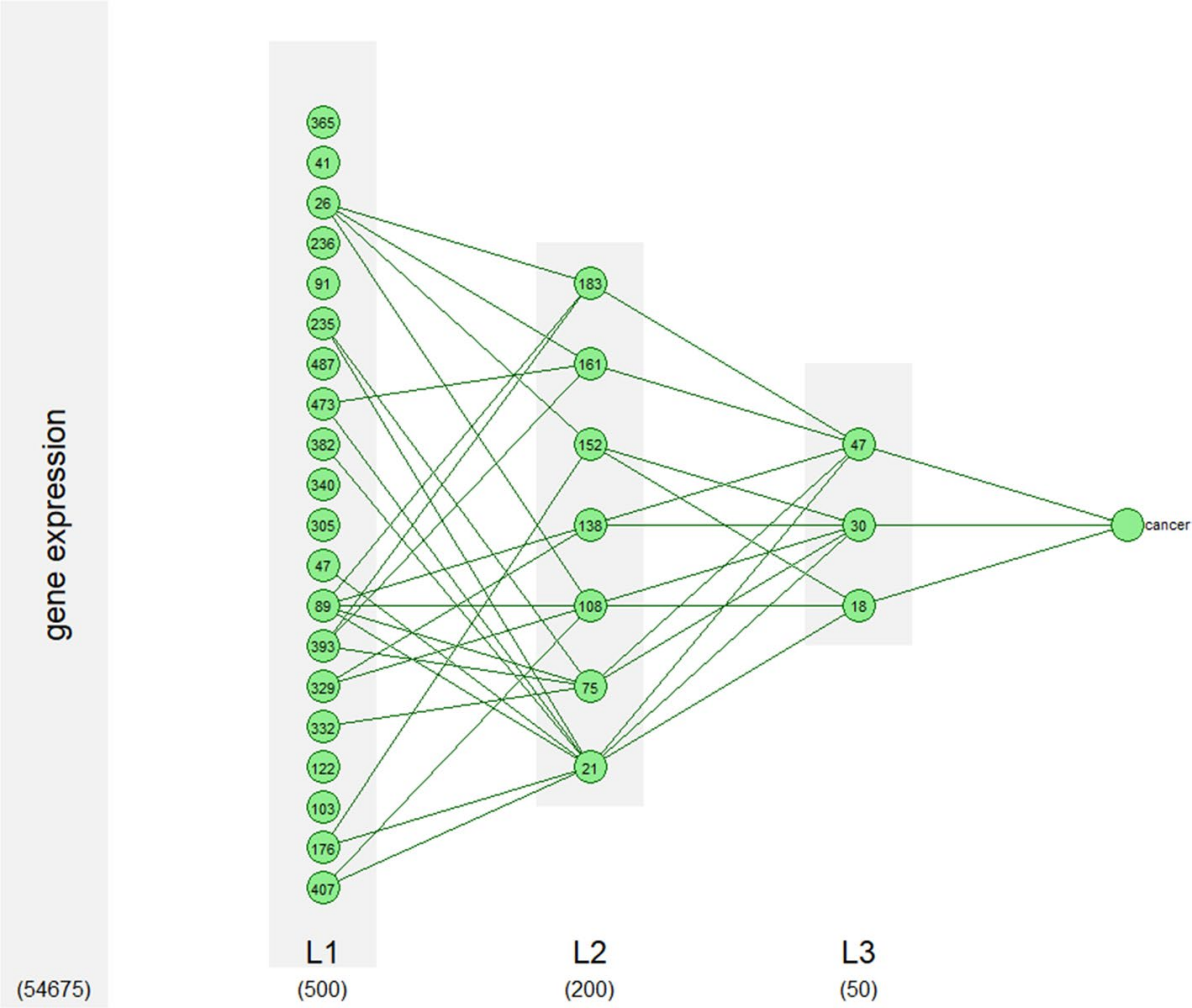
Significant neurons in the network for prediction of cancer

In this section, we illustrate the interest of our method by providing a biological interpretation of the neural network predicting cancer from the previous section. For each class, the important neurons of each layer are identified from the mean vector of the relevance scores by using the method described in section 5.3.1. We identified 20, 7 and 3 important neurons in hidden layers 1,2 and 3 respectively for cancer prediction and 26, 14 and 4 important neurons in hidden layers 1, 2 and 3 respectively for non-cancer prediction. With the same approach, we identified the most important connections (connections with the highest absolute value of relevance). Figure 5 shows the important neurons and connections in the network for cancer prediction (see the Additional file 1 for the non cancer prediction). We also identified more than 1000 important probes that can not be represented in the figure. For each of the important neurons, a list of genes, biological functions GO, metabolic pathways KEGG and diseases in DOLite is associated using the procedure described in 5.3.2 and 5.3.3. We now provide a biological interpretation of the important neurons and discuss their importance in the differentiation between cancer and non cancer patients. We opted to focus on few neurons of the relevance network, a more complete interpretation of an extracted subnetwork from the relevance network can be found in the Additional file 1.

The important neurons of layer 1 can be grouped into subgroups depending on the functions enriched among the important genes they contain. Overall, the enriched functions belonged to three main categories which are the cell cycle, metabolic processes, morphogenesis even though the three are linked. The cell cycle pathway is linked to cell polarity as well as to cell structure pathways in a complex system which is what was reproduced through our neural network. The first category which is the cell cycle category contains neuron 26. Neurons 26 is the only neuron that focuses solely on cell cycle pathways. It is associated with a list of 593 genes. The most enriched GO terms are mitotic cell cycle process, GO:1903047, and mitotic cell cycle, GO:0000278 with the other important enriched GO terms in this neuron all belonging to mitotic division and DNA replication. This neuron hence specialized in detecting genes in relation to cell proliferation, an essential element in cancer as cancer originates from uncontrollable growth of abnormal, mutated cells. The KEGG enrichment analysis of this neuron showed that among the most enriched metabolic pathways are the ones in which mitosis and DNA replication are involved such as cell cycle, which is the cell division cycle in general and the focal adhesion pathway which regulates the cell cycle pathway. In fact, in cancer, especially in the metastasis stage, the cancerous cells show alteration in their focal adhesion dynamics as cancerous cells will want to detach from their fixation site and move through the Extra-Cellular Matrix (ECM) to the blood and lymphatic vessels [25]. Other enriched pathways that are known to be important in cancer and linked to cell proliferation were also found enriched in this neuron such as PPAR signaling pathway [26], adipocytokine signaling pathway [27], p53 signaling pathway [28], homologous recombination and DNA replication. In order to go further in the analysis, we performed a DOLite analysis which consists of associating a disease to the genes that were linked to significant GO terms. We found 40 genes associated with cancer and 3 genes associated with advanced cancer. Among the 43 genes, we found some that are already known to be linked to multiple cancer types. The LEP gene, which is the gene responsible for making Leptin, was found to be linked to many cancers mainly breast cancer, colorectal cancer, hepatocellular cancer and thyroid cancer [29]. Another gene we found was BRCA1 which is already known to be one of the genes that can cause breast and ovarian cancer when mutated and that is now being linked to other types of cancer such as melanoma [30]. Included in the list are also the RAD51 and PCNA genes. The RAD51 gene is a pivotal homologous recombination gene and has already been found to be overexpressed in the following tumors: cervical cancer, non-small cell lung cancer, breast cancer, ovarian cancers, pancreatic cancer, melanoma and glioblastoma [31]. As for the PCNA gene, which has an important role in controlling DNA replication, it was found to be involved in many cancer types: in breast cancer, for example, PCNA methylation was found to be cancer specific [32].

In the second hidden layer of the network, we had 7 important neurons. Among them is neuron 183 which is one of the most significant neurons of layer 2. It was found to have an important link with neuron 26 of the first hidden layer. When looking at the GO terms enriched at this level, we see that the most enriched one is regulation of chromosome segregation, GO:0051983, which regulates the separation of the genetic material. Other enriched GO terms are also linked to chromosome segregation and cell division. As such, we can say that this neuron also specialized in cell division and the GO terms of this neuron are co-occurring with the terms of the neuron of the previous layer. However, when we look at the KEGG enriched pathways, we can see that only one pathway, which is the base excision repair pathway, was enriched. This pathway, as the name suggests, repairs any DNA damage that occurs during the cell cycle. Thus, we can say that this neuron is more specific than the one in the first layer. Mutations in the base excision repair pathway were found to be linked to cancer mainly prostate and lung cancer [33, 34]. For this neuron, we looked into the enriched genes using DOlite as we did with previous neurons in order to determine to which diseases they are associated. Among the 24 genes found to be involved in cancer, we found MSH6, which is a DNA mismatch repair gene, and ECT2, which is a gene guanine nucleotide exchange factor whom previous study showed that, along with other co-expressed genes, this gene is potentially involved in the base excision pathway. DOlite analysis also showed that there are 4 genes involved in esophageal cancer enriched in this neuron.

**Fig. 6:**
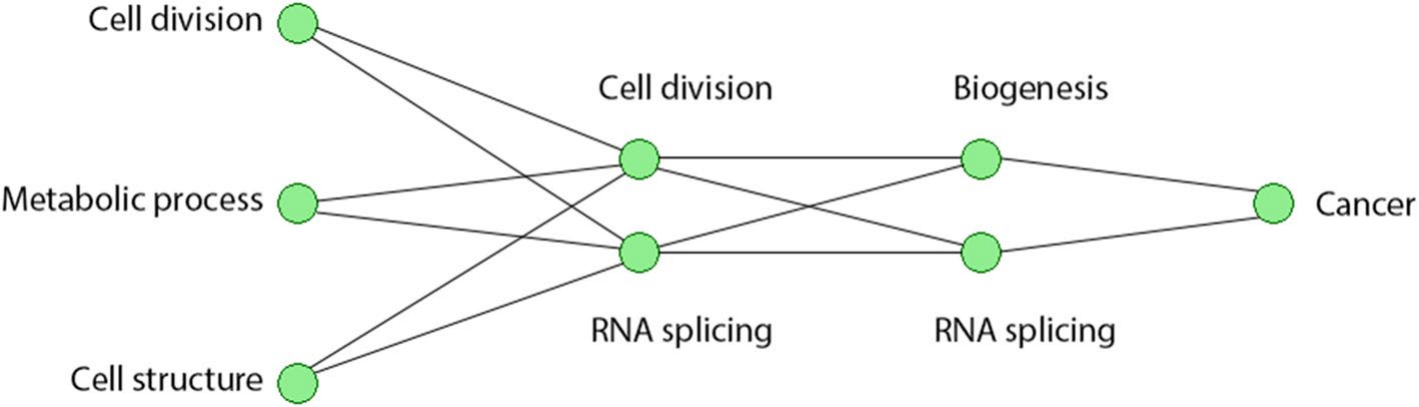
A simplified network showing the enriched pathways in each layer

In the third and final hidden layer of the model, we had only three significant neurons. Neuron 47 has mainly GO terms linked to RNA processing enriched. However, it is more specific as among the GO terms enriched are the ncRNA processing and rRNA processing. The KEGG enrichment analysis is in concordance with this, as the most enriched pathway is the RNA transport pathway. In summary, we can say that the important neurons we identified, focused mainly around the cell cycle and the pathways that co-occurs with it such as metabolic and RNA splicing processes. The first layer was the most general layer with little specializing. As we advanced in the layers, each neuron tended to specialize in an element of the cell cycle with the last layer having a neuron specific to non coding RNA processing (see Fig. 6).

## Discussion

We point out that the goal of the interpretation is to explain how the model works and not how biology works. Sometimes, there is no obvious relation between the biological functions or metabolic pathways, returned by the interpretation, and the predicted phenotype. This does not necessarily mean that the predictions are not reliable. Recall that a model looks for correlations between the output and the input and not for causalities. When a biological function, that is not related to the phenotype, is identified in the important neurons, it is possible that this function either has an indirect correlation or is linked by an unknown causality relation with the phenotype. Moreover, let us not forget that the biological databases used are not a perfect description of biology, but just a representation of current knowledge of biology. In these databases, a lot of information is missing because it is unknown and some available information may be incorrect.

We finish by giving the different types of conclusions that we can draw from a biological interpretation of a model. We identify three cases based on the results of the interpretation. In the first case, the majority of the elements provided by the biological interpretation are related to the predicted phenotype. This means that the model bases its predictions on elements consistent with the biological knowledge. This should improve the trust in the model in addition to its prediction performance. The second case is the opposite. Most parts of the elements provided by the biological interpretation are known to be unrelated to the predicted phenotype. Since the predictions are based on elements inconsistent with the current biological knowledge, the reliability of the model must be questioned. The model may overfit or be misled by a bias in the training set. In the last case, the biological interpretation mostly provides elements that may or may not be related to the predicted phenotype. Interpretation does not help to evaluate the trust that we can place in the model. However, in this case, we can use the interpretation as a tool for biological discovery. Since the model finds a relationship between the phenotype and gene expression based on the elements identified by the interpretation, we can assume that there is a link between these elements and the phenotype. Interpretation can therefore lead to new biological hypotheses to be investigated by biologists.

## Conclusion

In this paper, we propose an original approach for biological interpretation of deep learning models for phenotype prediction from gene expression data. Our main objective is to identify the neurons and inputs of the NN that contribute to the predictions and to link them to biological knowledge. The model is reduced to a sub-network containing the relevant connections and neurons involved in the prediction. These neurons are then associated with a list of genes and the corresponding biological knowledge (GO, KEGG, and DOLite). Our experiments, based on cancer prediction, show that (1) deep learning approach outperforms classic machine learning methods on large training datasets, (2) our approach produces interpretations more coherent with biology than the state-of-the-art based on WM approach, (3) we can provide a comprehensive explanation of the predictions for biologists and physicians. Ongoing work concerns additional biological analysis, comparison and validation that are necessary to get a comprehensive picture of the logic behind the neural network predictions. Future work concerns the introduction of biological knowledge inside the neural network in order to guide the learning phase of the model. This allows to learn known biological concepts and may lead to biological discoveries.

## Methods

We present the deep neural network architecture used for gene expression data and our biological interpretation approach. The gradient method for neural network interpretation is the Layer-wise Relevance Propagation (LRP), which is adapted to identify the most important neurons that lead to the prediction as well as the identification of the set of genes that activate these important neurons. Finally, the important neurons and genes are linked to the Gene Ontology (GO), the Kyoto Encyclopedia of Genes and Genomes (KEGG) and the Disease Ontology Annotation List (DOLite) in order to propose a biological interpretation of the neural network model.

### Deep multilayer perceptron

Given a classification task with *K* classes, a classifier is a function that associates a class to an input vector: $F:x \mapsto y$. In our work $x\in \mathbf {R}^{p}$ is a gene expression profile, $y \in \{c_1, \ldots ,c_K\}$ is the predicted class corresponding to the phenotype and *F* is a deep neural network. In the context of gene expression data, we use a multilayer perceptron architecture with *L* layers. In this architecture, the neurons are organized in layers where each neuron is connected to all neurons of the previous layer and all neurons of the next layer. The input layer receives the gene expression profiles, each neuron takes the expression of one probe. The output layer returns the probabilities to belong to each class (one neuron for each class). The activation of the i-th neuron of the layer *l* can be expressed as: $a_i^{(l)} = g \left( \sum _{j=1}^{n_{l-1}} a_j^{(l-1)} w_{ji}^{(l)} + b_i^{(l)} \right)$, where $w_{ji}^{(l)}$ is the weight of the connection from the j-th neuron of the layer ($l-1$) to the i-th neuron of the layer *l*, $b_i^{(l)}$ is the bias of the i-th neuron of the layer *l* and $n_l$ the number of neurons in the layer *l*. We denote $z_i^{(l)} = \sum _{j=1}^{n_{l-1}} a_j^{(l-1)} w_{ji}^{(l)} + b_i^{(l)}$ the input of the i-th neuron of the layer *l* and $z_{ji}^{(l)} = a_j^{(l-1)} w_{ji}^{(l)}$ the signal going from the j-th neuron of the layer $l-1$ to the i-th neuron of the layer *l*. The activation function, *g* , in this work, corresponds to the rectified linear unit function (ReLU) $g(z)=max\{0,z\}$ for the hidden layers and the softmax $g(z^{(L)})_k=exp(z_k^{(L)})/\sum _{j=1}^{K} exp(z_j^{(L)})$, where $z^{(L)} = \{z_1^{(L)},\ldots , z_K^{(L)}\}$, for the output layer. With these notations, the expression profile representing a patient at the input of the network is noted $a^{(0)}$, the posterior probability of each class $c_k$ estimated by the network is noted $a_k^{(L)}=g(z^{(L)})_k$ and the prediction of the neural network is $F(x)=argmax_k\{a_k^{(L)}\}.$

### Layer-wise relevance propagation (LRP)

The prediction of a patient’s phenotype is obtained by propagating its gene expression profile through the network and evaluating the neurons in the feed-forward pass. Gradient methods compute the influence of each variable and neuron of the network for a given prediction. Among all gradient methods presented in the “Introduction” section, we chose the LRP [18] for two reasons: firstly LRP produces results well aligned with human intuition [23], secondly it does not need reference inputs. The reference input, needed by some gradient based methods, is an input vector with no information, for example it corresponds to black pixels in images. For gene expression data it is difficult to justify the choice of such a reference. In our experiments, we noted that the choice of the gradient method does not significantly impact the interpretation since the results from different gradient methods are correlated for gene expression data and multilayer perceptron (see the Additional file 1).

**Fig. 7:**
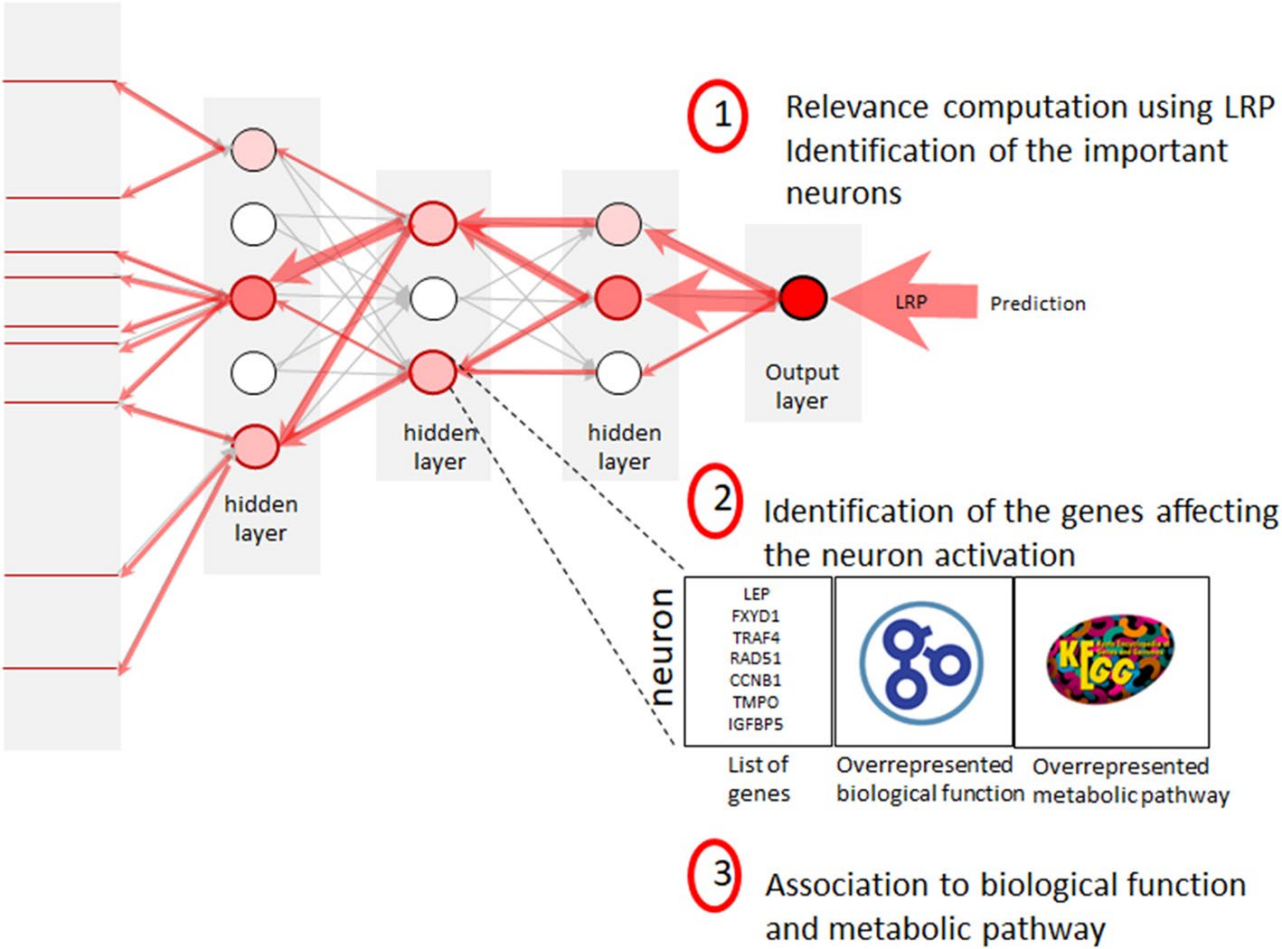
The biological interpretation of deep neural network approach

The idea of LRP is to backpropagate the signal of the output neuron of interest through the network. For a given output neuron, at each layer *l*, the relevance score of each neuron and each connection is computed from the relevance scores of the layer ($l+1$). These relevance values represent how the neurons and connections have contributed to the activation of the studied output neuron. This method is based on layer-wise conservation principle that forces the preservation of the propagated relevance between layers and neurons i.e. the sum of neuron relevance is constant through all layers and for each neuron the sum of output connection relevance is equal to the sum of input connection relevance. Assume that for a given patient, the network predicts the class $c_k$. To explain this prediction, we studied the *k*-th neuron of the output layer *L*. Let $R_k^{(L)}=z_k^{(L)}$ be the relevance of the output neuron to be backpropagated through the network. Note that we backpropagate $z_k^{(L)}$, that is the activation of the output neurons before applying the softmax activation function. The relevance of a neuron is propagated through all of its input connections (including the bias) proportionally to the signal passing in the connections.

The relevance of the connection from the i-th neuron of the layer *l* to the *j*-th neuron of the layer ($l+1$) is defined by:

1 $$\begin{aligned} R_{i \leftarrow j}^{(l)} = R_j^{(l+1)} \frac{z_{ij}^{(l+1)}}{z_j^{(l+1)}} \end{aligned}$$

The relevance of a neuron is defined by the sum of the relevances of all its output connections:

2 $$\begin{aligned} R_i^{(l)} = \sum _{j=0}^{n_{l+1}} R_{i \leftarrow j}^{(l+1)} = \sum _{j=0}^{n_{l+1}} R_j^{(l+1)} \frac{z_{ij}^{(l+1)}}{z_j^{(l+1)}} \end{aligned}$$

A drawback of these backpropagation formulas is that for small values $z_j$, relevances $R_i$ can take unbounded values. Unboundedness can be overcome by introducing a predefined stabilizer $\epsilon >0$ as follows:

3 $$\begin{aligned} R_{i \leftarrow j}^{(l)} = R_j^{(l+1)} \frac{z_{ij}^{(l+1)}}{z_j^{(l+1)} + \epsilon sign(z_j^{(l+1)})} \end{aligned}$$

When using this formula, the conservation principle is relaxed to overcome the numerical instability. The relevance is backpropagated following these formulas from one layer to another until it reaches the input (gene expression) layer.

Originally, LRP has been developed to interpret the prediction from images i.e. estimate the contribution of each pixel to the prediction of the class of a given image. In this work, we adapt and use LRP in the context of gene expression data. Moreover, our work focuses on the problem of model interpretation rather than prediction interpretation. Our analysis is therefore based on the average of relevances computed from a subset of the test sets and not on individual relevance scores. The LRP can also be used to explain the individual prediction but this analysis is out of the scope of this paper.

### Biological interpretation approach

The objective is to identify the biological functions and metabolic pathways that the neural network uses to predict each class. For each class the proposed interpretation approach can be decomposed into three steps (see Fig. 7). In the first step, we compute the relevance scores through the network and identify the most important neurons that allow predicting the class. Then, we associate with each important neuron a list of the significant genes affecting the neuron activation. Finally, biological functions, metabolic pathways and diseases are associated with each important neuron.

#### Selection of important neurons for predicted classes

The first step is to identify the neurons that most influence the predictions for each class. For this, we compute the relevance scores of all neurons for each prediction using the LRP procedure. These relevance scores associated with individual predictions are used to compute the model scores for each predicted class.

For each class $c_k$, a set of relevance values (one related to each patient predicted as belonging to the class $c_k$) is associated with each neuron. We call the mean of these relevance scores the average relevance of the neuron *i* in the layer *l* for the class $c_k$: $\hat{R}_{i,k}^{(l)}$. This average relevance score represents the influence of this neuron on the network to predict the class $c_k$. The most important neurons are the neurons with the highest absolute values of their average relevance score. To select the most important neurons, from each layer, we ranked the neurons according to their absolute average relevance scores and chose the most important ones. In our experiments, we empirically observed that the distribution of the average relevance scores is close to a Gaussian distribution centered at 0. Assuming that the average relevance scores follow a Gaussian distribution $N(0,\sigma _{k}^{(l)})$ where $\sigma _{k}^{(l)}$ is the empirical variance of the average relevance scores, we use the two-side t-test (p-value at 0.05 with Bonferroni correction) to determine the most extreme average relevance scores over the observed average relevance scores. We applied this procedure to identify the important neurons in each layer for a given class.

#### Association neurons-list of genes

LRP was conceived to associate relevant inputs to the prediction. In this step of our approach, we propose to associate with each important neuron the list of the genes that influence the activation of the neuron. For a given important neuron *i* in layer *l*, its activation $a_i^{(l)}$ is backpropagated using the LRP procedure in order to compute $R_j^{(0)}$, which is the relevance score of each input *j*. We then identify the most important inputs that have an impact on the activation of the neuron. As for the identification of important neurons, in our experiments, we used a two-side t-test to select the inputs related to the neuron since the observed distribution of the input relevance scores is close to a Gaussian distribution centered at 0. We can see the activation of a neuron as a non linear representation of the expression of the set of its associated genes. In order to associate each important neuron to a list of genes, we annotated the input with their GenBank accession number.

#### Biological interpretation of the neurons

The final step of our approach is to associate each important neuron to biological functions from Gene Ontology (GO), metabolic pathways from the Kyoto Encyclopedia of Genes and Genomes (KEGG) and diseases from Disease Ontology Annotation List (DOLite). For each neuron, we identify the over-represented GO functions in the list of genes associated to this neuron. We use a hypergeometric test to check if a GO function is over-represented in a neuron. Given a function $GO_i$, let $N_{GO_i}$ be the number of genes related to the function $GO_i$ and $N_G$ the number of genes present in the dataset. The probability of obtaining *T* genes related to the function $GO_i$ from a list of *M* random genes follows a hypergeometric law: $$p(T,M,N_G,N_{GO_i}) = \frac{ \left( {\begin{array}{c}N_{GO_i}\\ T\end{array}}\right) \left( {\begin{array}{c}N_G - N_{GO_i}\\ M-T\end{array}}\right) }{\left( {\begin{array}{c}N_G\\ M\end{array}}\right) }$$. The probability of obtaining at least *T* genes related to the function $GO_i$ in a random list of genes is defined by: $P(t \ge T) = \sum _{t=T}^{M}p(t,M,N_G,N_{GO_i})$. We considered that a Go function is over-represented in a list of genes when the probability $P(t \ge T)<0.05$. We applied the same procedure to identify the over represented metabolic pathway KEGG and related disease in a list of genes.

## Supplementary information

**Additional file 1**. Supplementary materials about (1) the comparison with WM interpretation, (2) the comparison of gradient methods, (3) the analysis of the relevance in function on the type of tissue, (4) the biological interpretation.

## Data Availability

All codes are available (https://entrepot.ibisc.univ-evry.fr/d/01c071dea2b64e55b406/). The datasets are available on the public microarray data repository ArryExpress (accession number E-MTAB-3732).

## Abbreviations

DOLite: Disease ontology annotation lite
GO: Gene ontology
KEGG: Kyoto encyclopedia of genes and genomes
LASSO: Least absolute shrinkage and selection operator
LRP: Layer relevance propagation
PCA: Principal component analysis
NN: Neural network
RF: Random forest
SVM: Support vector machines
WM: Weight mean
XGB: eXtreme gradient boosting
