# Supplementary material for: Biological interpretation of deep neural network for phenotype prediction based on gene expression
Source: BMC Bioinformatics. 2020 Nov 4;21:501. doi: 10.1186/s12859-020-03836-4 (PMC7643315; doi:10.1186/s12859-020-03836-4)
Supplement: Supplementary file 1 — Additional file 1. Supplementary materials about (1) the comparison with WM interpretation, (2) the comparison of gradient methods, (3) the analysis of the relevance in function on the type of tissue, (4) the biological interpretation. [file 12859_2020_3836_MOESM1_ESM.pdf]

Additional files for "Biological interpretation of deep neural network for phenotype prediction based on gene expression."

Full list of author information is available at the end of the article

Additional file 1: Comparison of gradient methods

Our approach uses a gradient method to estimate the impact of each gene and neuron on the predictions. In our experimentations we tested the main gradient method i.e. simple gradient [1], gradient×input method [2], integrated gradient LRP-ϵ and LRP-z. On the figure 1, for each pair of methods the dot plot and the correlation of the relevance scores from the two methods are given. The relevance scores given the the different methods are very correlated. All gradient methods will return almost the same important genes and neurons. Our interpretation approach is therefore not sensitive to the choice of the gradient method. The diagonal of the figure 1 shows the distribution of the relevance scores for each method. We observe that these distributions are very close to a Gaussian distribution, that validate the use of the two-sides t-test to select the most important genes and neurons.

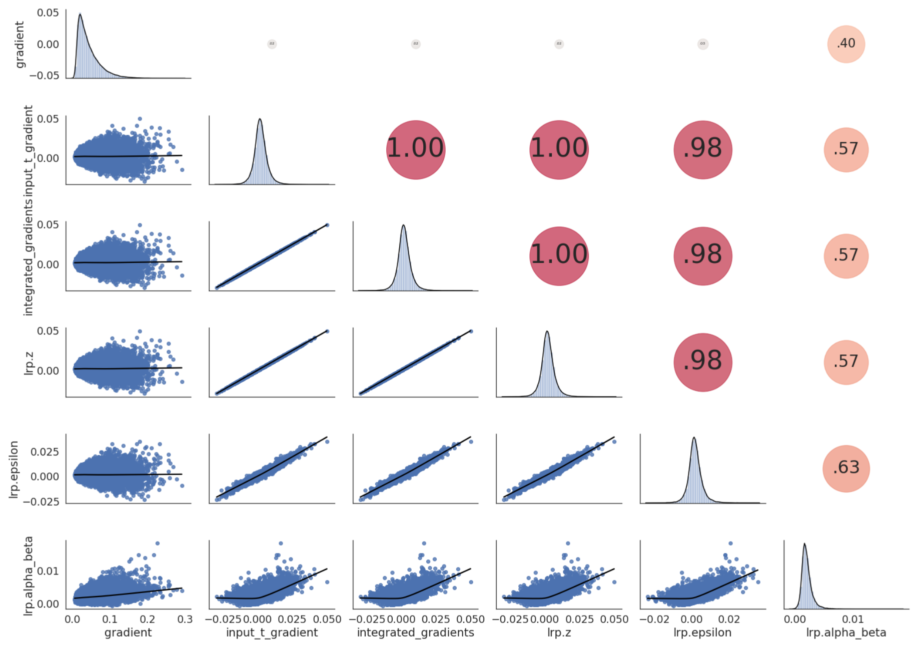

Figure 1 Comparison of relevance score of the different gradient methods. For each pair of methods a dot plot and the correlation of relevance is given. The diagonal shows the distribution of relevance of each methods.

Additional file 2: Analysis of the relevance score for non cancer prediction in function of the type of tissue

The figure 2 shows the hierarchical clustering of the test examples predicted as **non cancer** based on their relevance vector. In the first colored bar, red represents

prediction errors and green corresponds to good predictions. The next colored bars show the type of tissue. We observe the same pattern than in the dendrogram from examples predicted as cancer.

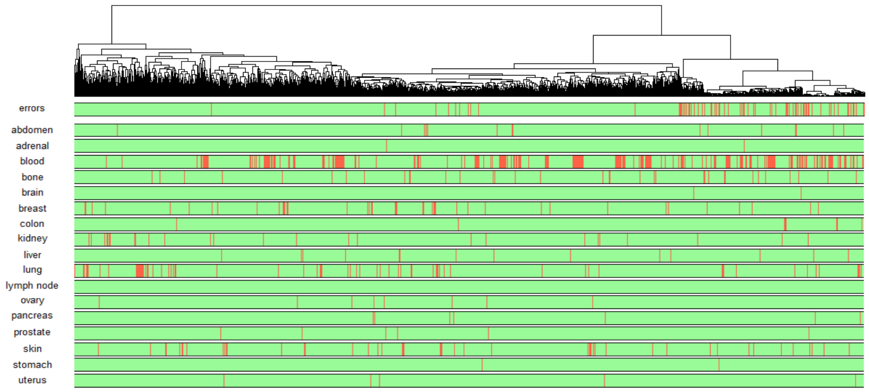

Figure 2 Hierarchical clustering of the test examples predicted as non cancer based on their relevance vector. Red leaf means a prediction error and green leaf means a good prediction.

Additional file3: Biological interpretation

We present in this section the complete biological interpretation of the network. The important neurons of layer 1 can be grouped into subgroups depending on the functions enriched among the important genes they contain. Overall, the enriched functions belonged to three main categories which are the cell cycle, metabolic processes, morphogenesis even though the three are linked. The cell cycle pathway is linked to cell polarity as well as to cell structure pathways in a complex system which is what was reproduced through our neural network. The first category which is the cell cycle category grouped two neurons which are neuron 26 and neuron 407. Neurons 26 is the only neuron that focus solely on cell cycle pathways. It is associated to a list of 593 genes. The most enriched GO terms are mitotic cell cycle process, GO:1903047, and mitotic cell cycle, GO:0000278 with the other important enriched GO terms in this neuron all belonging to mitotic division and DNA replication. This neuron hence specialized in detecting genes in relation to cell proliferation, an essential element in cancer as cancer originates from uncontrollable growth of abnormal, mutated cells. The KEGG enrichment analysis of this neuron showed that among the most enriched metabolic pathways are the ones in which mitosis and DNA replication are involved such as cell cycle, which is the cell division cycle in general and the focal adhesion pathway which regulates the cell cycle pathway. In fact, in cancer, especially in the metastasis stage, the cancerous cells show alteration in their focal adhesion dynamics as cancerous cells will want to detach from their fixation site and move through the Extra-Cellular Matrix (ECM) to the blood and lymphatic vessels ([3]. Other enriched pathways that are known to be important in cancer and linked to cell proliferation were also found enriched in this neuron such as PPAR signaling pathway [4], adipocytokine signaling pathway [5], p53 signaling pathway [6], homologous recombination and DNA replication. In order to go further in the analysis, we performed a DOLite analysis which consists of associating a disease to the genes that were linked to significant GO terms.

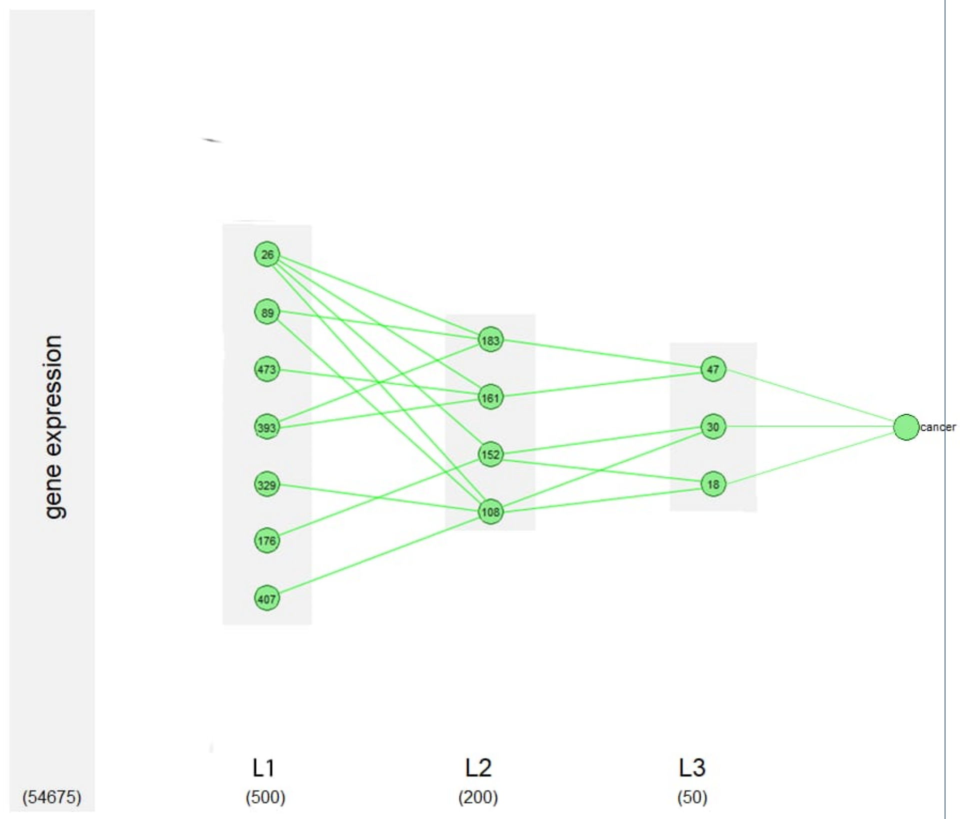

Figure 3 Sub network considered in the biological interpretation.

We found 40 genes associated to cancer and 3 genes associated to advanced cancer. Among the 43 genes, we found some that are already known to be linked to multiple cancer types (see figure 4). The LEP gene, which is the gene responsible for making Leptin, was found to be linked to many cancers mainly breast cancer, colorectal cancer, hepatocellular cancer and thyroid cancer [7]. Another gene we found was BRCA1 which is already known to be one of the genes that can cause breast and ovarian cancer when mutated and that is now being linked to other types of cancer such as melanoma [8]. Included in the list are also the RAD51 and PCNA genes. The RAD51 gene is a pivotal homologous recombination gene and has already been found to be overexpressed in the following tumors: cervical cancer, non-small cell lung cancer, breast cancer, ovarian cancers, pancreatic cancer, melanoma and glioblastoma [9]. As for the PCNA gene, which has an important role in controlling DNA replication, it was found to be involved in many cancer: in breast cancer, for example, PCNA methylation was found to be cancer specific [10]. Neuron 407 is associated to GO terms that are linked to cell motility with locomotion, GO:0040011, being the most enriched pathway which as we said before is expected in cancerous cells. This neuron also has enriched GO terms linked to the immune system. In addition, the most enriched KEGG terms are pathways in cancer as adherent pathways are listed among cancer pathways in KEGG as well as pathways belonging to cell migration and phagosome. We then looked at the DOlite analysis and found genes associated to primary tumors and to lymphoma,

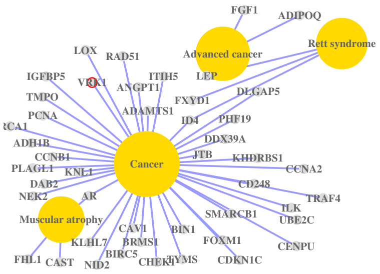

Figure 4 DOlite analysis results of the genes of neuron 26

explaining the presence of immune system enriched functions (see figure 5). Among the genes was the TPM1 gene which is a known tumor suppressor known to be downregulated in cancer [11]. Neuron 473 has enriched functions similar to this neuron. Another important neuron in the first layer is neuron 176 which belongs to

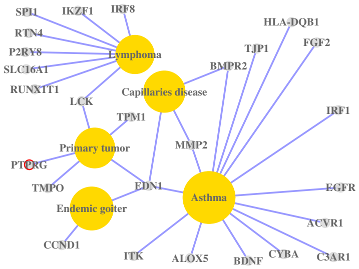

Figure 5 DOlite analysis results of the genes of neuron 407

the metabolic processes along with neuron 393. It is associated to 607 genes. The most enriched GO term is regulation of cellular metabolic process, GO:0031323, along with a multitude of other GO terms belonging to the cellular process family of GO terms as well as the metabolic process family. Hence, this neuron focused solely on capturing genes that are specific to metabolic processes. Metabolic processes group all chemical reactions and processes that are necessary to survive and live. Consequently, any alteration to those processes can lead to cancer. To better understand, we look closely into the KEGG enriched pathways and we find that the two most enriched pathways are pathways in cancer and MAPK signaling pathway with the latter being a part of the former. The different pathways involved in cancer as found by KEGG lead to tissue invasion and metastasis, proliferation, immortality and genetic damage among others. It can also lead to resistance to chemotherapy and can block differentiation. Other enriched pathways are pathways cancer specific pathways such as melanoma, endometrial cancer, prostate cancer and renal cell carcinoma. Neuron 235 is also another important neuron in the first layer. The most enriched GO term is cellular macromolecule catabolic process, GO:0044265, along with a multitude of other GO terms belonging to the catabolic process. Metabolic processes in general are crucial to cancer cell survival as they allow the cells to get the nutrients they need to proliferate. Catabolic processes, in particular, are important as they help degrade complex nutrients called macromelecules to replenish

the intracellular metabolic intermediates. In cancer cells, it is important to have catabolic degradation to keep an amino acids supply full [12]. Other than catabolic processes, GO terms linked to cell cycle and cell proliferation are also enriched. Afterwards, we performed a KEGG analysis to detect the most enriched pathways. As expected, the most enriched pathway is Ubiquitin mediated proteolysis. This system plays an important role in different cellular processes, including cell cycle and hence proliferation which is very important in cancer as we previously said and the system works by the degradation of the tagged protein by the 26S proteasome [13]. For example, it has been shown that this system regulates Myc destruction which controls the proliferation of cancerous cells ([14]). Other enriched pathways are p53 signaling pathway and apoptosis which are both in relation with the cell cycle pathway and are found among the pathways in cancer as classified in KEGG. As the enriched pathways are linked to cancer, we looked into the diseases associated to the genes of this list using DOlite and we found that there 31 genes that are associated with cancer (see figure 6). Among these genes, there are many that are already linked to cancer. BAX, a pro apoptotic gene, for example, is thought to be a direct target of p53, a tumor suppressor. With the majority of the tumors being linked to an inactivated p53 gene, this indicates that BAX is also repressed in cancer cells, thus, favoring their proliferation [15]. Another example is AKT2 which is an oncogene that was found overexpressed in some human ovarian and pancreatic carcinomas ([16]). Neurons 329 can be grouped under the both above categories

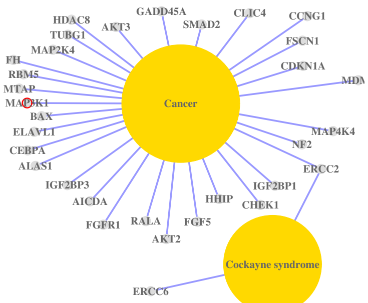

Figure 6 DOlite analysis results of the genes of neuron 235

as it contains enriched functions similar to all neurons discussed above. The last category contains neuron 89 which specializes in the environment of the cells. The enriched terms regulate the shape, angiogenesis and vascularization. In fact, after the division, cells will tend to set their environment, and irregularities in the shape can cause cancer whereas cancerous cells after division will to gain new blood vessels so that they can go from one site to another and undergo metastasis. In the second hidden layer of the network, we had 7 important neurons. Among them is neuron 183 which is one of the most significant neurons of layer 2 and it was found to have an important link with neuron 26 of the first hidden layer. When looking at the GO terms enriched at this level, we see that the most enriched one is regulation of chromosome segregation, GO:0051983, which regulates the separation of the genetic material. Other enriched GO terms are also linked to chromosome segregation and

cell division. As such, we can say that this neuron also specialized in cell division and the GO terms of this neuron are co-occurring with the terms of the neuron of the previous layer. However, when we look at the KEGG enriched pathways, we can see that only one pathway, which is the base excision repair pathway, was enriched. This pathway, as the name suggests, repairs any DNA damage that occurs during the cell cycle. Thus, we can say that this neuron is more specific than the one in the first layer. Mutations in the base excision repair pathway were found to be linked to cancer mainly prostate and lung cancer [17] [18]. For this neuron, we looked into the enriched genes using DOlite as we did with previous neurons in order to determine to which diseases they are associated. Among the 24 genes found to be involved in cancer, we found MSH6, which is a DNA mismatch repair gene, and ECT2, which is a gene guanine nucleotide exchange factor whom previous study showed that, along with other co-expressed genes, this gene is potentially involved in the base excision pathway. DOlite analysis also showed that there are 4 genes involved in esophageal cancer enriched in this neuron (see figure 7). Neuron 152 has

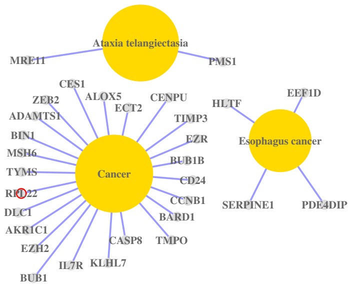

Figure 7 DOlite analysis results of the genes of neuron 183

372 significant genes. The GO enrichment analysis showed that the most enriched pathway is cell division, GO:0051301. Other enriched terms are related to mitosis and chromosome segregation. This neuron has important links to neurons 26 and 176 from the previous layer. As we previously said, neuron 26 is also specialized in the cell cycle and the GO terms of both neurons are co-occurring. In addition, neuron 176, as we already said, specialized in metabolic processes. Interestingly, it was found, in previous studies, that metabolic processes control cell cycle. In fact, in order for the cell to go through cell division, it requires the nutrients and energy that were produced by the different metabolic processes and any alteration to these processes can alter the cell cycle and hence favor cancer [19]. Thus, we can say that this neuron is also more specific than neuron 26 of the first layer and is some sort of continuation of the GO terms and pathways enriched in neuron 176. The KEGG enrichment analysis supports our finding with the most enriched pathway being cell cycle with 10 genes. In addition, in this layer, we have two other significant neurons: neurons 108 and 161 which also specialize in the interphase part of the cell cycle. In the third and final hidden layer of the model, we had only three significant neurons. Neuron 18 is the first neuron to be significant. As we go deeper in the neuron, we have less and less enriched GO terms. In this neuron, the few enriched GO terms were mainly focused on RNA processing, cell proliferation and biogenesis. RNA processing in few words, is the removal of non coding parts from pre-mRNA whereas

biogenesis refers to the production of proteins by ribosomes. We then look at the KEGG enriched pathways and we found that there are three pathways enriched. The most enriched one is Spliceosome which corresponds to the RNA splicing of pre-mRNA and hence choosing which isoform to form. Studies showed that mutations in RNA splicing genes can lead to the favoring of pro cancerous isoforms resulting in cancer-specific mis-splicing or for example can cause global alterations when not enough introns are removed [20]. The second enriched pathway is biogenesis, which as we said is the formation of proteins by ribosomes. However, in abnormal cases, which is in the case of the presence of oncogenes or the mutation of tumor suppressor genes, ribosomes start to produce a larger number of proteins which helps cancer grow [21]. The last enriched pathway is the MMR repair pathway which corresponds to the repair system that occur during DNA replication which is linked to the cell cycle. Mutations in this system, whether genetic (Lynch Syndrome or Constitutional MisMatch Repair Deficiency) or sporadic can cause a multitude of cancers with colorectal and endometrial being among the most frequent ones. We can say that although some genes in the list (3) were enriched for the MMR repair pathway but the majority of the other genes were enriched in the RNA processing and biogenesis which means that, in this layer, significant genes tended to be genes involved specifically and mainly in the DNA replication process and biogenesis. As we can see, as we go deeper into the neural network, the neurons tend to become more focused on a more specific area as compared to the previous layer that is more general. Neuron 30 is another one of the 3 important neurons of layer 3. In this neuron, the few enriched GO terms were mainly focused on RNA processing, cell proliferation and biogenesis. As we previously explained, these enriched GO terms are very crucial in cancer as they represent the different stages of the cell cycle. We also looked at the enriched KEGG functions of this neuron and we found that the most enriched pathways are RNA transport and spliceosome which, in the case of nuclear export of the mRNA to the cytoplasm, are both coupled. This neuron has important links with neurons 108 and 152 of the previous layer. The last neuron in this layer is neuron 47. This neuron similarly to the previous one, has mainly GO terms linked to RNA processing enriched. However, it is more specific as among the GO terms enriched are the ncRNA processing and rRNA processing. The KEGG enrichment analysis is in concordance with this, as the most enriched pathway is the RNA transport pathway. In brief, we can say that the important sub network we extracted, focused mainly around the cell cycle and the pathways that co-occurs with it such as metabolic and RNA splicing processes. The first layer was the most general layer with little specializing. As we advanced in the layers, each neuron tended to specialize in an element of the cell cycle with the last layer having a neuron specific to non coding RNA processing (see figure 8). We also compared neuron 235 between the cancer and non-cancer network as it is one of the significant neurons in both classes. Interestingly, we found that the enriched function in both cases do not belong to the same enriched KEGG pathways even though some of the enriched GO terms were in common (metabolic processes) suggesting that different pathways were used to differentiate between the two classes even in the scope of the same neuron.

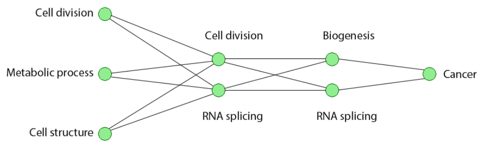

Figure 8 A simplified network showing the enriched pathways in each layer.

Author details

References

1. Baehrens, D., Schroeter, T., Harmeling, S., Kawanabe, M., Hansen, K., Müller, K.-R.: How to explain individual classification decisions. J. Mach. Learn. Res. **11**, 1803–1831 (2010)
2. Simonyan, K., Vedaldi, A., Zisserman, A.: Deep inside convolutional networks: Visualising image classification models and saliency maps. ArXiv e-prints (2013). 1312.6034
3. Maziveyi, M., Alahari, S.K.: Cell matrix adhesions in cancer: the proteins that form the glue. Oncotarget **8**(29), 48471 (2017)
4. Tachibana, K., Yamasaki, D., Ishimoto, K., et al.: The role of ppars in cancer. PPAR research **2008** (2008)
5. Li, J., Han, X.: Adipocytokines and breast cancer. Current problems in cancer **42**(2), 208–214 (2018)
6. Vazquez, A., Bond, E.E., Levine, A.J., Bond, G.L.: The genetics of the p53 pathway, apoptosis and cancer therapy. Nature reviews Drug discovery **7**(12), 979 (2008)
7. Dutta, D., Ghosh, S., Pandit, K., Mukhopadhyay, P., Chowdhury, S.: Leptin and cancer: Pathogenesis and modulation. Indian journal of endocrinology and metabolism **16**(Suppl 3), 596 (2012)
8. Mersch, J., Jackson, M.A., Park, M., Nebgen, D., Peterson, S.K., Singletary, C., Arun, B.K., Litton, J.K.: Cancers associated with brca 1 and brca 2 mutations other than breast and ovarian. Cancer **121**(2), 269–275 (2015)
9. Chen, Q., Cai, D., Li, M., Wu, X.: The homologous recombination protein rad51 is a promising therapeutic target for cervical carcinoma. Oncology reports **38**(2), 767–774 (2017)
10. Stoimenov, I., Helleday, T.: Pcna on the crossroad of cancer (2009)
11. Pugacheva, E.N., Roegiers, F., Golemis, E.A.: Interdependence of cell attachment and cell cycle signaling. Current opinion in cell biology **18**(5), 507–515 (2006)
12. DeBerardinis, R.J., Chandel, N.S.: Fundamentals of cancer metabolism. Science advances **2**(5) (2016)
13. Ciechanover, A., Orian, A., Schwartz, A.L.: Ubiquitin-mediated proteolysis: biological regulation via destruction. Bioessays **22**(5), 442–451 (2000)
14. Salghetti, S.E., Kim, S.Y., Tansey, W.P.: Destruction of myc by ubiquitin-mediated proteolysis: cancer-associated and transforming mutations stabilize myc. The EMBO journal **18**(3), 717–726 (1999)
15. Miyashita, T., Reed, J.C., *et al.*: Tumor suppressor p53 is a direct transcriptional activator of the human bax gene. Cell **80**(2), 293–300 (1995)
16. Ruggeri, B.A., Huang, L., Wood, M., Cheng, J.Q., Testa, J.R.: Amplification and overexpression of the akt2 oncogene in a subset of human pancreatic ductal adenocarcinomas. Molecular Carcinogenesis: Published in cooperation with the University of Texas MD Anderson Cancer Center **21**(2), 81–86 (1998)
17. Trzeciak, A.R., Nyaga, S.G., Jaruga, P., Lohani, A., Dizdaroglu, M., Evans, M.K.: Cellular repair of oxidatively induced dna base lesions is defective in prostate cancer cell lines, pc-3 and du-145. Carcinogenesis **25**(8), 1359–1370 (2004)
18. Baudot, A., De La Torre, V., Valencia, A.: Mutated genes, pathways and processes in tumours. EMBO reports **11**(10), 805–810 (2010)
19. Kalucka, J., Missiaen, R., Georgiadou, M., Schoors, S., Lange, C., De Bock, K., Dewerchin, M., Carmeliet, P.: Metabolic control of the cell cycle. Cell cycle **14**(21), 3379–3388 (2015)
20. Dvinge, H., Kim, E., Abdel-Wahab, O., Bradley, R.K.: Rna splicing factors as oncoproteins and tumour suppressors. Nature Reviews Cancer **16**(7), 413 (2016)
21. Pelletier, J., Thomas, G., Volarević, S.: Ribosome biogenesis in cancer: new players and therapeutic avenues. Nature Reviews Cancer **18**(1), 51 (2018)
